# Downregulation of DJ-1 Fails to Protect Mitochondrial Complex I Subunit NDUFS3 in the Testes and Contributes to the Asthenozoospermia

**DOI:** 10.1155/2018/6136075

**Published:** 2018-04-03

**Authors:** Yupeng Wang, Yi Sun, Xin Zhao, Renpei Yuan, Hui Jiang, Xiaoping Pu

**Affiliations:** ^1^National Key Research Laboratory of Natural and Biomimetic Drugs, Peking University, Beijing 100191, China; ^2^Department of Molecular and Cellular Pharmacology, School of Pharmaceutical Sciences, Peking University, Beijing 100191, China; ^3^Department of Urology, Peking University Third Hospital, Beijing 100191, China

## Abstract

Asthenozoospermia (AS), an important cause of male infertility, is characterized by reduced sperm motility. Among the aetiologies of AS, inflammation seems to be the main cause. DJ-1, a conserved protein product of the *PARK7* gene, is associated with male infertility and plays a role in oxidative stress and inflammation. Although our previous studies showed that a reduction in DJ-1 was accompanied by mitochondrial dysfunction in the sperm of patients with AS, the specific mechanism underlying this association remained unclear. In this study, we found that compared to the patients without AS, the expression of mitochondrial protein nicotinamide adenine dinucleotide dehydrogenase (ubiquinone) Fe-S protein 3 (NDUFS3) was also significantly decreased in the sperm of patients with AS. Similarly, decreased expression of DJ-1 and NDUFS3 and reduced mitochondria complex I activity were evident in a rat model of AS. Moreover, we showed that the interaction between DJ-1 and NDUFS3 in rat testes was weakened by ORN treatment. These results suggest that the impaired mitochondrial activity could be due to the broken interaction between DJ-1 and NDUFS3 and that downregulation of DJ-1 in sperm and testes contributes to AS pathogenesis.

## 1. Introduction

Asthenozoospermia (AS) is a common cause of human male infertility [1], characterized by reduced sperm motility (grade A + B sperm motility < 50% or A < 25%). It is involved in more than 40% of infertility in men [2]. Inflammation is a largely reversible cause of male infertility. Neutrophils and macrophages can damage spermatozoa by generating proinflammatory cytokines and reactive oxygen species (ROS), which leads to oxidative stress and DNA fragmentation that may affect sperm motility and metabolism, consequently causing infertility [3, 4]. Mitochondria have been proposed as major contributors to oxidative stress, which can result in defective sperm in humans [5]. Electron microscopy showed that sperm from patients with AS have disordered mitochondria, with significantly shorter midpieces and fewer mitochondrial gyres than their normozoospermic counterparts [6]. Furthermore, sperm quality, particularly motility, is positively correlated with the enzymatic activity of electron transfer chain (ETC) complexes [7, 8] and the expression of ETC subunits [9]. Mitochondrial protein nicotinamide adenine dinucleotide dehydrogenase (ubiquinone) Fe-S protein 3 (NDUFS3) is a core component of complex I (CI) in the respiratory chain of the mitochondrial matrix for CI assembly and activity [10] and was found to be associated with DJ-1 in NIH3T3 and HEK293 cells [11].

DJ-1, the *PARK7* gene product, is a ubiquitous protein of 189 amino acids that belongs to the Thi/PfpI protein superfamily of molecular chaperones. It is highly conserved in a variety of mammalian tissues, and mutations or deletions in *PARK7* have been found associated with many diseases, including male infertility. DJ-1 has been proposed to function as a survival factor and antioxidant [12, 13]. Furthermore, the function of DJ-1 in inflammation has been elucidated. DJ-1 regulates the expression of proinflammatory cytokines by regulating NF-*κ*B transcriptional activity in macrophages [14]. Additionally, DJ-1 has been shown to play a pivotal modulatory role by triggering inflammation and subsequently enhancing the secretion of IL-6 and TNF-*α* in liver progenitor cells [15].

DJ-1 and its homologues, sperm protein 22 (SP22) and contraception-associated protein 1 (CAP1), were the first proteins found to be correlated with male infertility [16–18]. DJ-1 is expressed after second spermatocytes appear during spermatogenesis and is ultimately mainly located in the sperm head, suggesting an important function for DJ-1 in spermatogenesis [17, 18]. Downregulation of DJ-1 was found in ejaculated sperm from Chinese patients with AS, and DJ-1 was shown to translocate into sperm mitochondria during oxidative stress to maintain mitochondrial structure [19, 20].

Ornidazole- (ORN-) treated rats have been a common animal model to study AS in the last several decades [21]. This model is established by daily intragastric administration of ORN to rats for 10~14 days [22]. Although DJ-1 has been found to be associated with NDUFS3 in some somatic cells, this interaction in male germ cells has not yet been reported, and its expression during spermatogenesis in AS is still unknown. In this study, we analyzed changes in the expression of and interaction between DJ-1 and NDUFS3 in sperm and testes to understand AS pathogenesis.

## 2. Material and Methods

### 2.1. Ethics Statement

#### 2.1.1. Participants

We enrolled 10 males (aged 21~45 years) diagnosed with infertility in the Department of Andrology, Peking University Third Hospital, Beijing, China, and 10 age-matched control subjects with normal semen parameters. Semen samples of patients with pyospermia or varicocele or with a history of smoking were not obtained for this study. All participants provided informed consent for participation in the study, and the study was approved by the Ethics Institutional Review Board of Peking University Third Hospital, under protocol number 2011SZ016.

#### 2.1.2. Animals

Sexually mature male Sprague-Dawley rats weighing 330~370 g at the beginning of the experiment were obtained from Charles River Laboratories Inc. (SCXK (Jing) 2012-0001, Beijing, China). The rats were maintained at a controlled temperature (24 ± 2°C) and housed by group in separate cages (12 h light/dark cycle) with access to food and water ad libitum. All experimental procedures involving the use of animals were approved by the Animal Care and Use Committee of Peking University.

### 2.2. Experimental Procedures with Human Sperm

#### 2.2.1. Western Blotting

Total sperm protein was extracted as described previously [19]. In brief, the sperm were washed twice with Hanks balanced salt solution with 4.2 g/L hydroxyethyl piperazine ethanesulfonic acid (HEPES), 0.35 g/L NaHCO_3_, 0.9 g/L d-glucose, 0.1 g/L sodium pyruvate, 0.025 g/L soybean trypsin inhibitor, and freshly added EDTA-free halt protease inhibitor cocktail (Thermo Fisher Scientific Inc., Rockford, USA) by centrifugation (2500 ×g, 10 min). The combined sperm pellets, resuspended in 80 mM Tris-HCl buffer (pH 7.4) with 150 mmol/L NaCl, 2 mmol/L ethylenediamine tetraacetic acid, 0.4 mmol/L dithiothreitol, and 0.1% sodium dodecyl sulfate (SDS), were ultrasonically oscillated and then homogenized for 1 h on ice. Finally, the mixtures were centrifuged at 12,000 ×g for 20 min to remove insoluble debris, and the supernatant fraction was frozen at −80°C until use. The protein concentration was measured using an enhanced bicinchoninic acid (BCA) assay kit (Beyotime, Shanghai, China). Proteins (60 *μ*g) were separated by 10% SDS-polyacrylamide gel electrophoresis (PAGE) and transferred to a polyvinylidene difluoride (PVDF) membrane (Millipore, Billerica, USA). Membrane was blocked with 5% nonfat milk in Tris-buffered solution with Tween-20 under agitation. Proteins were sequentially incubated with anti-NDUFS3 monoclonal antibody (1 : 3000, Abcam, Cambridge, UK) and horseradish peroxidase- (HRP-) conjugated IgG (1 : 5000, KPL, Gaithersburg, USA). GAPDH was used as an internal standard. Protein bands were visualized using an enhanced chemiluminescence detection kit (ECL; Applygen Technologies Inc., Beijing, China) and a Molecular Imager ChemiDoc XRS+ Imaging System (Bio-Rad, Hercules, USA). Quantity One software (Bio-Rad, Hercules, USA) was used to quantify the optical density of each band, and the amount of NDUFS3 was normalized to that of GAPDH.

#### 2.2.2. Immunofluorescence Assay

Spermatozoa were collected by density gradient centrifugation, using Percoll solution (Sigma, St. Louis, USA) as a medium [23]. All Percoll solutions were buffered with Biggers, Whitten, and Whittingham medium (BWW). Following centrifugation, sperm that accumulated at the bottom of the tube were collected.

According to the operation manual recommended by Life Technologies Corporation, spermatozoa were resuspended in prewarmed (37°C) BWW containing 200 nM MitoTracker Deep Red FM (MT-DR FM) (Invitrogen, Carlsbad, USA) and incubated at 37°C for 45 min. After staining, the spermatozoa were washed, spotted on glass slides, and air-dried. These preparations were fixed with 4% paraformaldehyde and then permeabilized with ice-cold acetone. After blocking with 10% goat serum, spermatozoa were sequentially incubated with anti-DJ-1 monoclonal antibody (1 : 200, Abcam, Cambridge, UK) or anti-NDUFS3 monoclonal antibody (1 : 200, Abcam, Cambridge, UK) and then goat anti-rat lgG secondary antibody (1 : 200, Alexa Fluor 555 conjugate) or goat anti-mouse lgG secondary antibody (1 : 200, Alexa Fluor 488 conjugate) (Thermo Fisher Scientific Inc., Rockford, USA). Normal rabbit or mouse IgG was used as a negative control. Nuclei were counterstained with Hoechst 33342. All samples were observed by laser scanning confocal microscopy (TCS SP8-Confocal-MP-FLIM, Leica, Mannheim, Germany).

### 2.3. Experimental Procedures with Rat Samples

#### 2.3.1. Rat Model of AS

A rat model of AS was generated by intragastric administration of ORN, according to a previously described method with some modifications [24]. Briefly, twenty sexually mature (330~370 g) male Sprague-Dawley rats were randomly assigned to two groups (ORN and control) with ten rats per group. In the ORN group, ORN was dissolved in 1% (*w*/*v*) sodium carboxymethylcellulose (CMC-Na) in water, and a dose of 400 mg/kg body weight was fed to adult male rats once a day by oral gavage for a period of 14 days. The control rats received 1% (*w*/*v*) CMC-Na in water without ORN throughout the experiment.

#### 2.3.2. Sperm Motility and Count

After the last intragastric administration of ORN or the control solution on day 14, the animals were anesthetized with intraperitoneal (i.p.) administration of 0.6% pentobarbital sodium (10 mL/kg). To assess sperm motility, sperm in the cauda epididymides were collected and prepared as described elsewhere [25]. In brief, each caudal epididymis was placed in 4 mL of saline prewarmed to 34°C and then incised in several places to allow the semen to ooze out. After this, an aliquot of 10 *μ*L was transferred to a histological slide. Under a light phase contrast microscope (200x magnification, Binocular, Olympus IX71, Tokyo, Japan), 400 sperm were analyzed and classified into three types on the basis of their motility: progressive motility (PR), nonprogressive motility (NP), and immotility (IM). Sperm motility data were expressed as the percentage of sperm that were progressively motile.

Sperm counts were evaluated according to an established method [26]. Semen obtained from cauda epididymides was diluted, thoroughly mixed, and transferred to a hemocytometer with a cover slip overlay. The semen was observed under a light microscope at 200x, and the sperm in each specimen were counted.

#### 2.3.3. Western Blotting

Rats were deeply anesthetized with 0.6% pentobarbital sodium (10 mL/kg i.p.) and cardiac perfused with normal saline. The testes and sperm were immediately homogenized in ice-chilled lysis RIPA buffer. The following steps were the same as those used for patients' samples. *β*-Actin was used as an internal standard. The standardized ratio of DJ-1 and NDUFS3 to *β*-actin band density was used to indicate change in expression.

#### 2.3.4. Sperm Mitochondrial Membrane Potential (MMP) Analysis

Spermatozoa collected from cauda epididymides were resuspended in prewarmed (37°C) phosphate-buffered saline (PBS) containing 200 nM MT-DR FM and incubated at 37°C for 45 min. After staining, the sperm were pelleted again by centrifugation and resuspended in prewarmed PBS. This spermatozoon suspension was spotted on glass slides and air-dried. The nuclei of cells on the slides were counterstained with Hoechst 33342 and mounted with antifade mounting medium. All samples were observed by laser scanning confocal microscopy (TCS SP8-Confocal-MP-FLIM, Leica, Mannheim, Germany).

#### 2.3.5. CI Enzyme Activity Assay

The analysis of CI enzyme activity in sperm and testes was performed using a CI Enzyme Activity Microplate Assay Kit (Abcam, Cambridge, UK) following the kit protocol. In short, CI was extracted and introduced into a microplate. After incubation at room temperature for 3 h, the microplate was washed, and assay solution was added. Absorbances of samples were tested at 450 nm for 30 min with an interval of 20 s, following the kinetic program in a FlexStation 3 Multi-Mode Microplate Reader (Molecular Devices, Sunnyvale, USA). Activity was expressed as the change in absorbance per minute per amount of sample loaded into a well. The data are shown as the standardized ratio of the activity in the ORN-treated rats to that in the control animals.

#### 2.3.6. Coimmunoprecipitation (Co-IP)

Using the method described above, the testes of rats were removed and homogenized in a glass homogenizer. The protein extract was preincubated with Protein A/G PLUS-Agarose beads (Santa Cruz Biotechnology) and normal goat IgG (M&C Gene Technology Ltd.) to remove nonspecifically adhered proteins. One milliliter of the above tissue lysate (approximately 500 *μ*g total cellular protein) was transferred to a microcentrifuge tube, and goat antibody to DJ-1 (1 : 100, Abcam, Cambridge, UK) was added for co-IP. Another 1.0 mL of lysate with normal goat IgG was used as the negative control group. The samples were incubated for 4 h at 4°C, and then Protein A/G PLUS-Agarose was added to each. After incubation on a rotating device at 4°C overnight, the immunoprecipitates were collected by centrifugation. The pellets were then washed and resuspended in electrophoresis sample buffer. After boiling, the samples were centrifuged to obtain the supernatant for SDS-PAGE analysis.

### 2.4. Statistical Analysis

Statistical analyses were performed with GraphPad Prism 6 for Windows. All data were expressed as means ± standard errors of the mean (SEM). One-way analysis of variance (ANOVA) was used to make comparisons between groups. *P* values < 0.05 were considered indicative of significant differences.

## 3. Results

### 3.1. General Parameters of Semen from Participants

General parameters, including age of participants, ejaculate volume, sperm density, and percentage of grade A and grade A + B sperm, are listed in Table 1. Ten males (aged 21~45 years), diagnosed with infertility as described above, were enrolled, and 10 age-matched males with normal semen parameters were registered as control subjects. No participants exhibited pyospermia or varicocele or had a history of smoking.

### 3.2. Change in NDUFS3 Expression in Sperm of Participants with AS

**Figure 1:**
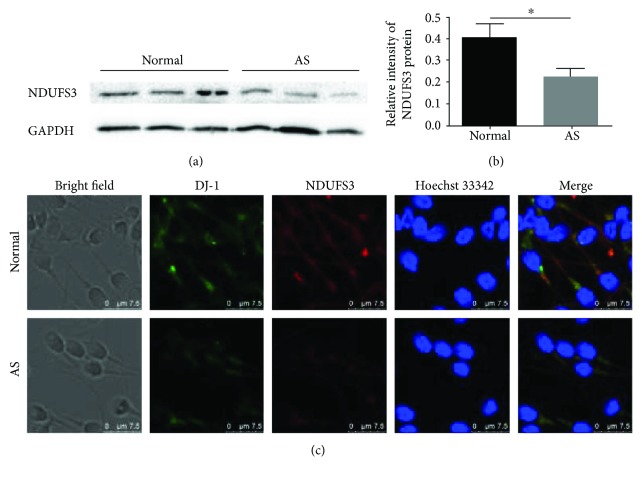
Downregulation of NDUFS3 in the sperm of patients with AS. (a) Representative immunoblot showing NDUFS3 expression in human sperm. (b) Quantification of “a.” NDUFS3 expression was normalized to that of GAPDH as a loading control. The results showed that NDUFS3 expression was reduced significantly in the sperm of patients with AS. (c) Immunofluorescence of DJ-1 (green) and NDUFS3 (red) in sperm from patients with and without AS. Sperm nuclei were stained with Hoechst 33342 (blue). Note that NDUFS3 were both downregulated in the sperm of patients with AS and their colocalization in the midpiece of sperm. ^∗^*P* < 0.05, one-way ANOVA test, *n* = 10 in each group. Scale bar = 7.5 *μ*m. AS: asthenozoospermia.

Our previous studies confirmed the significant reduction in DJ-1 in the sperm of patients with AS [19, 20]. NDUFS3 was identified as a single band of approximately ~30 kDa by Western blot (Figure 1(a)). Quantification of the NDUFS3 bands revealed that NDUFS3 expression decreased along with DJ-1 expression in patients with AS compared to that in control subjects (NDUFS3/GAPDH: 0.40 ± 0.07 versus 0.23 ± 0.04, *P* < 0.05; Figure 1(b)). In the immunofluorescence images, we found that both DJ-1 and NDUFS3 were expressed mostly in the midpiece of sperm (Figure 1(c)). These results suggested that the expression of DJ-1 and NDUFS3 correlated positively with low motility in AS sperm.

### 3.3. MMP Analysis in the Sperm of Participants

**Figure 2:**
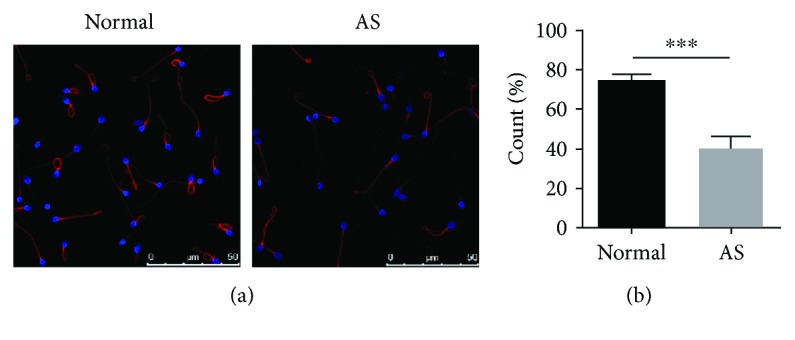
Decreased MMP in the sperm of patients with AS. (a) Immunofluorescence of mitochondria in human sperm, shown by MT-DR FM. Mitochondria in the midpiece of sperm were dyed red. Sperm nuclei were stained with Hoechst 33342 (blue). (b) Percentage MT-DR FM-positive sperm. Note that the sperm of patients with AS showed a significant reduction in MMP. ^∗∗∗^*P* < 0.001, one-way ANOVA test, *n* = 10 in each group. Scale bar = 50 *μ*m. AS: asthenozoospermia; MT-DR FM: MitoTracker Deep Red FM.

In human sperm, the mitochondrial sheath is organized in a helix of approximately 13 gyres surrounding the axoneme in the midpiece. Mitochondria in sperm were stained by MT-DR FM (Figure 2(a)). The percentage of MT-DR FM-positive sperm was measured in each of the two groups (72.13 ± 5.05% for normal samples, 42.90 ± 7.08% for AS samples, Figure 2(b)). The AS group contained a lower percentage of MT-DR FM-positive sperm than that in the normal group (*P* < 0.001), indicating that mitochondrial function was damaged in patients with AS.

### 3.4. Sperm Motility and Concentration in the AS Rat Model

**Figure 3:**
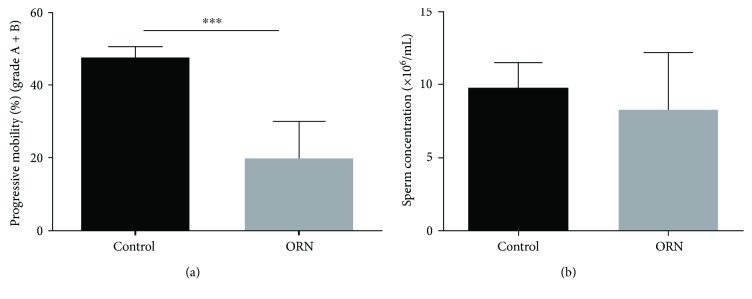
Analysis of sperm motility and concentration in control and ORN-treated rats. (a) Progressive motility (grade A + B) of sperm. The percentage of sperm with progressive motility was quantified and is showed in the histogram. Note that ORN induced a statistically significant reduction in the progressive motility of sperm. (b) Sperm concentration. There was no difference in the effect of ORN treatment on sperm concentration between the two groups. ^∗∗∗^*P* < 0.001, one-way ANOVA test, *n* = 10 rats in each group.

Sperm motility and concentration were analyzed to determine whether the AS rat model was successfully established. A statistically significant decrease in the proportion of sperm with progressive motility was found in the ORN-treated rats (19.8 ± 3.3%) compared to that in the control rats (47.7 ± 3.1%, *P* < 0.001; Figure 3(a)). Moreover, we found that ORN had no influence on rat sperm concentrations (Figure 3(b)). These results showed that ORN-treated rats displayed characteristics of AS [27, 28].

### 3.5. Reduced Expression of DJ-1 and NDUFS3 in AS Rats

**Figure 4:**
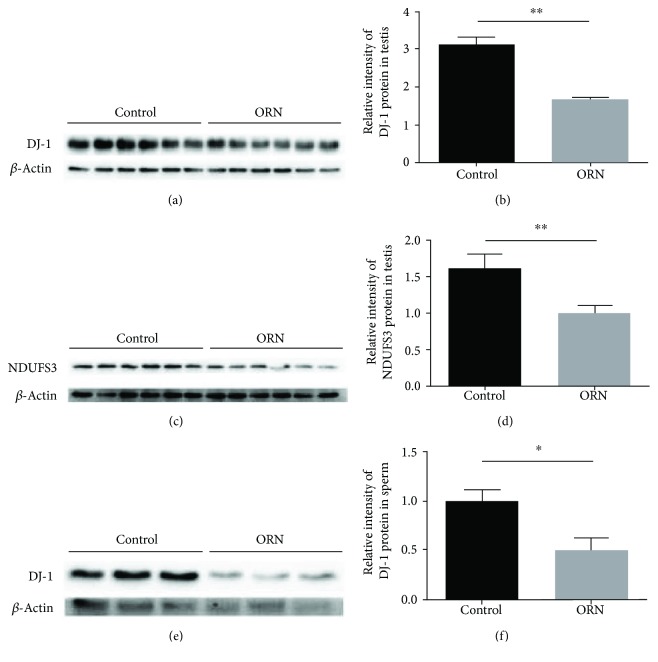
Decreased expression of DJ-1 and NDUFS3 in ORN-treated rat testes and sperm. (a) Western blotting detection of DJ-1 protein expression in rat testes. (b) Expression of DJ-1 was normalized against that of *β*-actin. Note that there was a reduction in DJ-1 in the testes from ORN-treated rats. (c) Western blotting detection of NDUFS3 protein expression in rat testes. (d) Expression of NDUFS3 was normalized against that of *β*-actin. These results showed that the expression of NDUFS3 decreased significantly in the testes of ORN-treated rats compared to that in control subjects. (e) Western blotting detection of DJ-1 protein expression in rat sperm. (f) Expression of DJ-1 was normalized against that of *β*-actin. These results showed that the expression of DJ-1 decreased significantly in the sperm of ORN-treated rats compared to that in control subjects. ^∗^*P* < 0.05, ^∗∗^*P* < 0.01, one-way ANOVA test, *n* = 6 rats in each group.

To investigate whether ORN-treated AS in rats was associated with DJ-1 and NDUFS3 expression during spermatogenesis, we examined DJ-1 and NDUFS3 expression levels in AS and control rats. In the Western blotting analysis, DJ-1 was identified as a single band at ~20 kDa (Figure 4(a)). Quantification of the DJ-1 bands revealed that the level of DJ-1 was reduced in the testes of AS rats (1.66 ± 0.07) compared to those in control rats (3.13 ± 0.18, *P* < 0.01; Figure 4(b)). Western blotting analysis detected DJ-1 as a single band at ~30 kDa in the rat testes (Figure 4(c)). The relative intensity of NDUFS3 protein was also deceased in the testes of ORN-treated rats (1.00 ± 0.11) compared to that in the control group (1.62 ± 0.20, *P* < 0.05; Figure 4(d)). DJ-1 was also identified as a single band at ~20 kDa in rat sperm (Figure 4(e)). The relative intensity of DJ-1 protein was decreased in the sperm of ORN-treated rats (0.50 ± 0.12) compared to that in the control group (1.00 ± 0.12, *P* < 0.05; Figure 4(f)). These results indicated that DJ-1 and NDUFS3 were affected by ORN treatment during spermatogenesis in rat sperm and testes.

### 3.6. Deceased MMP in the Sperm of AS Rats

**Figure 5:**
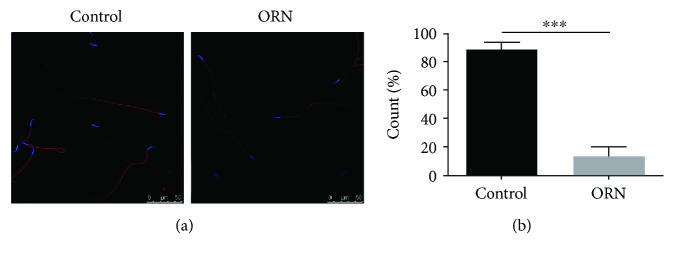
Decreased MMP in the sperm of ORN-treated rats. (a) Immunofluorescence of mitochondria in rat sperm, shown by MT-DR FM. Mitochondria in the midpiece of sperm were dyed red, and sperm nuclei were stained with Hoechst 33342 (blue). (b) Histogram showing the percentage of MT-DR FM-positive sperm. Note that the sperm in ORN-treated rats showed a significant reduction in MMP. ^∗∗∗^*P* < 0.001, one-way ANOVA test, *n* = 3 rats in each group. Scale bar = 50 *μ*m. MT-DR FM: MitoTracker Deep Red FM.

Similar to the results of the MMP assay of human samples, the mitochondria in the midpiece of sperm showed different amounts of staining (Figure 5(a)). An analysis of the images showed that the percentage of MT-DR FM-positive sperm in ORN-treated rats (88.85 ± 4.81) was statistically significantly reduced compared to that in the control group (13.75 ± 6.49, *P* < 0.001; Figure 5(b)), suggesting mitochondrial dysfunction in the sperm of AS rats.

### 3.7. Depressed CI Activity in the Sperm and Testes of AS Rats

**Figure 6:**
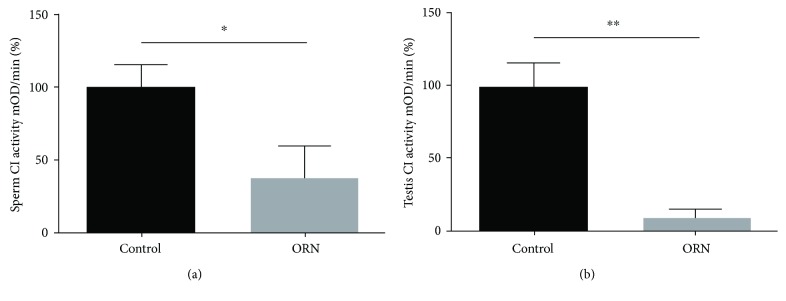
Decreased CI enzyme activity in the testes and sperm of ORN-treated rats. (a) Analysis of the CI activity of sperm in control and ORN-treated rats determined by CI enzyme activity microplate assay kit. CI enzyme activity in sperm was expressed in mOD/min. The histogram shows the ratio of the normalized mOD/min of rats in the ORN group compared to that in the control group. Note that CI activity in AS rat sperm was reduced relative to that in the control subjects. (b) Analysis of CI activity in testes of rats in the control and AS groups. The data indicate that CI activity in the testes of ORN-treated rats decreased significantly compared to that in the control group. ^∗^*P* < 0.05, ^∗∗^*P* < 0.01, one-way ANOVA test, *n* = 6 rats in each group. CI: complex I.

Mitochondrial CI is the first catalytic system in the respiratory chain. A specific reduction in mitochondrial CI activity inhibits sperm motility by regulating the NAD^+^/NADH redox balance. Thus, we tested CI enzyme activity in sperm and testes of AS rats using an assay kit. The CI activity in the sperm of AS rats (37.93 ± 21.51) was significantly reduced compared to that in the control rats (100.0 ± 15.92, *P* < 0.05; Figure 6(a)). The CI activity in the testes of AS rats showed the same tendency toward decrease (9.91 ± 5.54), unlike that observed in normal rats (100.0 ± 16.97, *P* < 0.05; Figure 6(b)). The data showed reduced mitochondrial activity in the sperm and testes of AS rats.

### 3.8. Weakened Interaction between DJ-1 and NDUFS3 in Rat Testes

**Figure 7:**
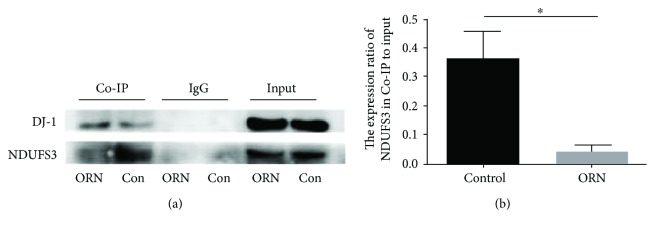
Direct interactions between DJ-1 and NDUFS3 demonstrated by coimmunoprecipitation. (a) The protein extracts were immunoprecipitated with an anti-DJ-1 antibody or IgG (negative control), and the precipitates were analyzed by Western blotting with anti-DJ-1 and anti-NDUFS3 antibodies. (b) The final coimmunoprecipitation result was obtained by subtracting the amount obtained from that of the negative control. The histogram shows the amount of DJ-1-NDUFS3 complex (presented as the ratio of the amount of normalized coimmunoprecipitated NDUFS3 versus that in the input) in rat testes. Note that DJ-1 and NDUFS3 were shown to interact in rat testes, and the ability of these two proteins to bind was significantly decreased in the testes of ORN-treated rats. ^∗^*P* < 0.05, one-way ANOVA test, *n* = 3 rats in each group.

To assess the endogenous association of DJ-1 with the mitochondria complex, proteins from the rat testes were immunoprecipitated with anti-DJ-1 antibody, and the precipitates were analyzed by Western blotting with anti-NDUFS3 and anti-DJ-1 antibodies. Western blotting analysis identified DJ-1 as a single band at ~20 kDa and NDUFS3 as a single band at ~30 kDa (Figure 7(a)). The result confirmed NDUFS3 to be a DJ-1-binding protein in the rat testes. Moreover, the interaction between these proteins was weakened in the AS rat testes (0.37 ± 0.095) compared to that in normal rats (0.046 ± 0.022, *P* < 0.05; Figure 7(b)). Thus, DJ-1, an integral mitochondrial protein, appears to play a role in maintaining CI activity, in conjunction with NDUFS3, in the rat testes.

## 4. Discussion

In the present study, we found that the expression of NDUFS3 and DJ-1 was both decreased in the sperm of patients with AS. We established an AS rat model to investigate this association further. CI activity was decreased in the testes and sperm of AS rats. DJ-1 and NDUFS3 expression levels were similarly reduced in the AS rat testes. The expression of DJ-1 was decreased in AS rat sperm. Moreover, a protein interaction between DJ-1 and NDUFS3 was demonstrated in the rat testes for the first time, and this interaction was weakened in AS.

Although the exact etiology of diminished sperm motility is still generally unexplained, ultrastructural defects of the sperm flagellum due to congenital defects and sperm degeneration caused by genital infections, oxidative stress, anti-sperm antibodies, cryopreservation, or metabolic disorders have been implicated [29]. Among the possible causes of AS, mitochondrial dysfunction in sperm is one of the most relevant causes, because sperm need large amounts of energy for mobility of their flagella and activity during fertilization. The generation of cellular energy for sperm motility can be accomplished through both oxidative phosphorylation and glycolysis in various regions of the sperm flagellum [30]. MMP is a key parameter that represents mitochondrial function. Its decrease can imply the disruption of the mitochondrial electron transport chain, which results in cellular dysfunction and even death [31, 32]. Our results showed mitochondrial dysfunction in the sperm of patients with AS. Several other researchers have found defects in mitochondrial respiratory activity in idiopathic as well as varicocele-related cases of AS [33, 34]. Very recently, less motile sperm from infertile patients were shown to exhibit lower MMP. With an increase in sperm MMP, sperm motility and fertility potential also increase [35]. In our study, ORN-treated rat sperm as well as testes showed decreased mitochondrial activity compared to that in the control group, suggesting that mitochondrial dysfunction in AS sperm might occur in the testes during spermatogenesis.

DJ-1, a protein related to male reproduction and infertility, has pleiotropic functions, ranging from a role as a chaperone with protease activity to that of a transcriptional regulator, redox sensor, and antioxidant scavenger [36]. In 1997, DJ-1 was first identified as a putative oncogene product that transformed mouse NIH3T3 cells in cooperation with activated ras [37]. Studies conducted in rats and mice have shown that DJ-1 expression is highly correlated with male infertility. When exposed to sperm toxicants such as ORN or epichlorohydrin, male rats and mouse showed reversible infertility, with reduced sperm motility and decreased expression of DJ-1 in sperm and epididymides [17, 38]. The fact that DJ-1 can serve as a biomarker for male fertility in both of these animals and in humans has been noted previously [39]. In 2011, downregulation of DJ-1 in sperm ejaculated from patients with AS was identified in a proteomic study comparing normal motile human sperm and that in idiopathic AS [19, 40]. Another study suggested that oxidation modification of DJ-1 was intensified in sperm from patients with AS compared to that in subjects without AS [20]. The distribution of DJ-1 in ejaculated sperm is in the surface of the posterior part of the head, the anterior part of the midpiece, and spermatozoa flagella. Our study showed that downregulation of DJ-1 may occur during spermatogenesis because decreased levels of DJ-1 were observed in the testes of AS rats by Western blotting. And the localization of DJ-1 in the midpiece of sperm indicated its function in mitochondria of sperm. These findings suggest that DJ-1 plays an essential role in sperm motility and AS.

Mitochondria generate adenosine triphosphate by oxidative phosphorylation (OXPHOS) via the mitochondrial respiratory chain, which consists of five multisubunit complexes (CI–V) composed of at least 75 nuclear DNA-encoded and 13 mitochondrial DNA- (mtDNA-) encoded proteins [41]. A high correlation between sperm motility and the activity of some mitochondrial enzymes (citrate synthase and respiratory CI, II, I + III, II + III, and IV) has been found [42]. NDUFS3 is a poorly characterized component of CI in the mitochondrial respiratory chain that is localized in the matrix portion of this multisubunit complex. Cleavage of NDUFS3, caused by lymphocyte protease granzyme A (GzmA), can lead to the production of ROS and ultimately cell death [43]. This study is the first to show the involvement of NDUFS3 in AS. We discovered reduced NDUFS3 expression in the sperm of patients with AS and in the testes of AS rats for the first time. This phenomenon suggests that a reduction in NDUFS3 in the testes and sperm may be to blame for low sperm motility. The reduced expression of NDUFS3 may inhibit the normal function of mitochondrial CI in spermatogenic cells of the testes, leading to decreased CI activity and sperm motility in AS.

The similar trends in the expression of NDUFS3 and DJ-1 in the sperm of patients with AS and the testes of AS rats indicated a correlation between these proteins in germ cells. In fact, colocalization of DJ-1 and NDUFS3 has been shown in NIH3T3 and HEK293 cells [11]. However, mitochondria in sperm are significantly different from those in somatic cells. The difference between mitochondria in sperm and somatic cells is reflected in both their morphology and biochemistry [44]. In our study, the protein interaction between DJ-1 and NDUFS3 was first confirmed in rat testes by coimmunoprecipitation. Moreover, this interaction was reduced in AS. Decreased expression of DJ-1 after knockdown of *PARK7* results in reduced CI activity in cells [45], and numerous studies have supported the role of DJ-1 in protecting cells against oxidative stress and maintaining mitochondrial structure. Recently, DJ-1 was found to translocate into sperm mitochondria in conditions of oxidative stress in Chinese patients with AS [20]. Thus, mitochondrial dysfunction caused by decreased levels of DJ-1 in sperm is a possible etiology of AS. DJ-1 can maintain mitochondrial function during oxidative stress by working with Pink1/Parkin pathway. An increase in mitochondrial DJ-1, regulated by Pink1/Parkin, can reduce ROS-induced damage in mitochondria [46]. Protection against mitochondrial damage of DJ-1 protein is probably promoted by the oxidation of C106, a cysteine residue in DJ-1 [47]. The correlation of DJ-1 and NDUFS3 suggests that NDUFS3 is a target of CI that assists DJ-1 in protecting mitochondria function. Decreased expression of DJ-1 as well as reduced DJ-1 and NDUFS3 binding in AS may lead to the loss of DJ-1 protection of CI, ultimately reducing sperm motility.

Animal models, historically, have played a critical role in the exploration and characterization of disease pathophysiology, identification of drug targets, and evaluation of novel therapeutic agents and treatments in vivo [48]. In this study, we established a classical ORN-treated rat model to investigate the function of DJ-1 and NDUFS3 in AS. The significantly reduced sperm motility and unchanged sperm concentration exhibited in this rat model conformed to observations in typical AS [22]. Although the AS rat model has been used in other studies, the results obtained here should be verified in the testes of humans. In addition, the sample size and types of human sperm samples used in this study were limited, and this study should be expanded to different types of AS pathogenesis.

## 5. Conclusions

In summary, we have shown a statistically significant reduction in DJ-1 and NDUFS3 expression in patients with AS and in a rat model. Interactions between DJ-1 and NDUFS3 in the testes were demonstrated and suggest that DJ-1 may play a role in maintaining mitochondrial function by means of the association with NDUFS3 during spermatogenesis in the testes. This protective function may be weakened in AS because of a reduction in binding ability as well as a decrease in the amount of DJ-1. This study suggests that downregulation of DJ-1 and NDUFS3 expression likely contributes to mitochondrial dysfunction, which may underlie AS pathogenesis, since current treatments for AS involve in vitro fertilization techniques rather than treatment of male infertility. These findings contribute to a deeper understanding of mitochondrial function in spermatogenesis in AS and may lead to the identification of a new therapeutic target for drug discovery.

## Figures and Tables

**Table 1:** General seminal parameters.

	Age	pH	Ejaculate volume (mL)	Sperm density (10^6^/mL)	Grade A sperm (%)	Grade A + B sperm (%)
Control	29.90 ± 1.233	7.420 ± 0.08406	4.140 ± 0.4110	57.23 ± 6.306	37.75 ± 1.984	60.39 ± 2.951
AS	30.30 ± 1.221	7.390 ± 0.05859	3.640 ± 0.4031	59.53 ± 11.85	11.95 ± 1.753^∗∗∗∗^	22.47 ± 2.139^∗∗∗∗^

^∗∗∗∗^ *P* < 0.0001, one-way ANOVA test, *n* = 10 in each group.
